# Complement in Thrombotic Microangiopathies: Unraveling Ariadne's Thread Into the Labyrinth of Complement Therapeutics

**DOI:** 10.3389/fimmu.2019.00337

**Published:** 2019-02-27

**Authors:** Eleni Gavriilaki, Achilles Anagnostopoulos, Dimitrios C. Mastellos

**Affiliations:** ^1^BMT Unit, Hematology Department, G. Papanicolaou Hospital, Thessaloniki, Greece; ^2^Division of Biodiagnostic Sciences and Technologies, INRASTES, National Center for Scientific Research Demokritos, Athens, Greece

**Keywords:** thrombotic microangiopathy, complement inhibitors, hemolytic uremic syndrome, HELLP syndrome, transplant-associated thrombotic microangiopathy

## Abstract

Thrombotic microangiopathies (TMAs) are a heterogeneous group of syndromes presenting with a distinct clinical triad: microangiopathic hemolytic anemia, thrombocytopenia, and organ damage. We currently recognize two major entities with distinct pathophysiology: thrombotic thrombocytopenic purpura (TTP) and hemolytic uremic syndrome (HUS). Beyond them, differential diagnosis also includes TMAs associated with underlying conditions, such as drugs, malignancy, infections, scleroderma-associated renal crisis, systemic lupus erythematosus (SLE), malignant hypertension, transplantation, HELLP syndrome (hemolysis, elevated liver enzymes, and low platelets), and disseminated intravascular coagulation (DIC). Since clinical presentation alone is not sufficient to differentiate between these entities, robust pathophysiological features need to be used for early diagnosis and appropriate treatment. Over the last decades, our understanding of the complement system has evolved rapidly leading to the characterization of diseases which are fueled by complement dysregulation. Among TMAs, complement-mediated HUS (CM-HUS) has long served as a disease model, in which mutations of complement-related genes represent the first hit of the disease and complement inhibition is an effective and safe strategy. Based on this knowledge, clinical conditions resembling CM-HUS in terms of phenotype and genotype have been recognized. As a result, the role of complement in TMAs is rapidly expanding in recent years based on genetic and functional studies. Herein we provide an updated overview of key pathophysiological processes underpinning complement activation and dysregulation in TMAs. We also discuss emerging clinical challenges in streamlining diagnostic algorithms and stratifying TMA patients that could benefit more from complement modulation. With the advent of next-generation complement therapeutics and suitable disease models, these translational perspectives could guide a more comprehensive, disease- and target-tailored complement intervention in these disorders.

## Introduction

Thrombotic microangiopathies (TMAs) represent a heterogeneous group of syndromes with the same phenotype: a clinical triad of microangiopathic hemolytic anemia (MAHA), thrombocytopenia and organ damage. This heterogeneous group of syndromes with considerable clinical overlap includes two major entities with distinct pathophysiology: thrombotic thrombocytopenic purpura (TTP) and hemolytic uremic syndrome (HUS) (1). Besides these two well-defined clinical conditions, the TMA spectrum also includes pathologies associated with underlying conditions, such as drugs, malignancy, scleroderma-associated renal crisis, systemic lupus erythematosus (SLE), malignant hypertension, transplantation, HELLP syndrome (hemolysis, elevated liver enzymes, and low platelets), and disseminated intravascular coagulation (DIC).

Since clinical presentation alone is not sufficient to differentiate between these entities, pathophysiological features need to be used for early diagnosis and appropriate treatment. Over the last decades, our understanding of the complement system has evolved rapidly leading to the characterization of diseases fueled by complement dysregulation that are also referred to as “complementopathies” (2). These are disorders in which activation of the complement system is a driving factor in disease pathophysiology and with evidence of effective complement inhibition in the disorder.

Among TMAs, atypical HUS has long served as an archetypal disease model of complement dysregulation, in which mutations of complement-related proteins represent the first hit of the disease and complement inhibition is an effective and safe strategy. Based on this knowledge, conditions resembling atypical HUS in terms of phenotype and genotype have emerged. As a result, the role of complement in TMAs is rapidly expanding in recent years due to genetic and functional studies (3). In an effort to facilitate early diagnosis and treatment, two recently published consensus documents have changed the terminology of these syndromes from an underlying disease-based model to a pathophysiology-driven model (4, 5). This review is based on the standardization of terminology proposed for TMAs by Scully et al. (5). Among others, this consensus has introduced the term complement-mediated HUS (CM-HUS) to describe discrete and also overlapping clinical entities with pronounced microangiopathic and thrombophilic manifestations which are shared by HUS and are likely underpinned by genetic alterations and/or functional derailment of the complement system leading to inflammatory damage of the glomerular endothelium (5).

Realizing the unmet needs of better understanding TMAs in this complex setting, this review aims to summarize current knowledge regarding complement activation in TMAs focusing on (a) complement-mediated HUS (b) infection-associated HUS, (c) HELLP syndrome, and (d) transplant-associated TMA. Emphasis will be placed on defining the clinical features that will enable complement modulation using new and emerging therapeutic options.

### Pathophysiology of Complement Activation

#### Complement-Mediated Hemolytic Uremic Syndrome (CM-HUS)

##### Clinical features

Diagnosis of complement-mediated HUS (CM-HUS) remains a clinical diagnosis of exclusion. Although it has been traditionally considered a pediatric disease, onset occurs in adulthood for the majority of patients (6). The syndrome manifests with signs and symptoms of anemia, thrombocytopenia and acute kidney injury. Other complications have been also reported, including neurologic, pulmonary and gastrointestinal disorder, peripheral gangrene, arterial stenosis, dilated cardiomyopathy and cardiorespiratory arrest.

Differential diagnosis of CM-HUS requires exclusion of secondary causes of TMAs, such as DIC, drugs, malignancy, scleroderma-associated renal crisis, SLE. Box 1 summarizes differential diagnosis in CM-HUS. In addition, shiga-toxin testing is necessary to exclude infection-associated or typical HUS that will be further discussed. Then, the differential diagnosis lies between TTP and HUS, with ADAMTS13 (a disintegrin and metalloproteinase with thrombospondin type 1 motifs, member 13) activity being the only reliable clinical diagnostic tool. In patients with ADAMTS13 activity less than 10%, diagnosis of TTP is established. TTP is a TMA caused by impaired processing of ultra large von Willebrand factor multimers due to severe deficiency of ADAMTS13. Severe ADAMTS13 deficiency is either inherited (Upshaw-Schulman syndrome) (7, 8) or acquired and immune-mediated, resulting from autoantibodies directed against ADAMTS13 (9–11). Detection of ADAMTS13 inhibitors suggests that the disorder has an immune-mediated background. Due to disease pathophysiology, plasma exchange is an effective treatment of immune-mediated TTP that needs to be employed immediately (12, 13).

Box 1 **Differential diagnosis of TMAs.**

**1. ADAMTS13 deficiency**

Thrombotic thrombocytopenic purpura

**2. Complement dysregulation**

Complement-mediated hemolytic uremic syndrome

**3. Infection associated**

Shiga-toxin

Campylobacter jejuni

Streptococcus pneumonia

Human immunodeficiency virus

Cytomegalovirus

Epstein–Barr virus

Parvovirus B19

BK virus

Influenza

**4. Disseminated intravascular coagulation**

**5. Systemic lupus erythematosus**

**6. Antiphospholipid antibody syndrome**

**7. Scleroderma**

**8. Vasculitis/glomerulonephritis**

**9. Pregnancy**

**10. Malignant hypertension**

**11. Drugs**

Calcineurin or mTOR inhibitors

Quinine

Estrogen/progesterone

Gemcitabine/mitomycin C

Interferon

Vascular endothelial growth factor inhibitors/tyrosine kinase inhibitors

Cocaine

**12. Metabolic/cell signaling**

Cobalamin responsive methylmalonic acidemia

Diacylglycerolkinase epsilon mutation

**13. Malignancy**

**14. Transplantation**

Based on this knowledge, common clinical criteria for CM-HUS include: (1) a serum creatinine level at or above the upper limit of the normal range, (2) microangiopathic hemolytic anemia, (3) thrombocytopenia, (4) ADAMTS13 activity of 5% or more, (5) and negative stool tests for Shiga toxin–producing infection (14). The majority of clinical studies in the field have adjusted these criteria to certain cut-off values to define HUS (15).

##### Functional evidence of complement activation

The wide appreciation that deregulated AP-mediated complement activation, both in the fluid phase and on the glomerular endothelial cell surface, has a definitive pathogenic role in CM-HUS and likely in other HUS-like TMAs, has been supported by a series of genetic studies and mechanistic investigations that have revealed fascinating roles of complement-derived effectors, as either disease drivers or exacerbators, in HUS-related pathologies.

The pathogenic role of complement in driving HUS-related pathologies is tightly linked to the distinct anatomical blueprint of the glomerular capillary network and the unique complement-activating properties and AP regulatory potential of the endothelial surface in the kidney, as compared to the microvasculature of other organs such as the brain, heart, or lung (16). The ability of endothelial surfaces to withstand autologous complement attack is also associated with the integrity of the sugar-rich glycocalyx that overlays these cells and its ability to recruit endogenous complement regulators, such as complement factor H (CFH), through their binding to polyanionic glycosaminoglycans comprising this specialized layer. In diseases such as CM-HUS where vascular endothelial/platelet activation disturb the homeostatic control of the endothelium, gradual deterioration of the glycocalyx through a sequence of thrombogenic and inflammatory insults exposes the underlying endothelial cell layer to a derailed intravascular complement system. This is particularly relevant to CM-HUS pathology as approximately two thirds of patients harbor mutations in complement genes, including loss-of-function variants in complement regulators such as membrane cofactor protein (MCP), CFH, and complement factor I (CFI) (17, 18). Loss of surface expression of MCP on endothelial cells, in conjunction with complement-activating insults such as the release of heme during mechanical hemolysis in the occluded vessels, appear to drive a vicious cycle of AP amplification and C3b deposition on the endothelial surface. The prevalent concept is that genetic defects in complement regulation in these patients shape a predisposing phenotype toward complement overactivation. This “at-risk” complement phenotype is then coupled to a secondary “hit” that can propagate complement activation in the kidney microvasculature (e.g., heme release), thus exacerbating the disease and fueling a vicious cycle that culminates in complement exhaustion and glomerular damage (19, 20). Notably, the clinical overlap between TTP and CM-HUS has nurtured the hypothesis that the susceptibility of the glomerular endothelium to complement attack might also be modulated by intrinsic determinants, such as the expression of soluble factors that promote platelet aggregation and microthrombi formation in the vasculature. In this direction, it has been suggested that the increased susceptibility of glomerular endothelial cells to complement activation might partly be attributed to the reduced release of von Willebrand Factor (vWF) which promotes complement deposition on the glomerular endothelial cells (21).

The strong penetrance of complement gene variants in CM-HUS is supported by genetic studies that have revealed a pathogenic role for certain complement haplotypes harboring mutations in C3, MCP, complement factor B (CFB) and CFH (22–28). Interestingly, certain C3 mutations in CM-HUS patients have been shown to code for gain-of-function C3 variants that form stable, decay-resistant C3 convertases, through high-affinity binding to CFB and conversely, through reduced binding to regulators that mediate their breakdown, such as MCP, CFH or complement receptor 1 (CR1), e.g., R139W/C3 mutant (26, 28). Of note, such disease-associated C3 variants are thought to drive HUS pathology, in the context of an overall compromised complement regulatory landscape (i.e., presence of at-risk CFH and MCP haplotypes), and consequent to a priming event that makes the renal endothelium more susceptible to C3 deposition and inflammatory damage (26, 29). Consistent to this notion, recent functional and *in silico* prediction studies have identified a number of gain-of-function CFB genetic variants that predispose for an overactive AP though stabilization of the C3 convertase, C3bBb, and increased resistance to decay by regulators such as FH (30). However, these findings cannot be generalized to all complement–related HUS/ TMA cases and caution should be exercised when attempting to classify such rare variants as disease-causing factors.

Several *in vitro* models have been utilized to demonstrate effects of complement activation in experimental studies. Endothelial cells play the central role in these models as the basic target cells of complement-induced damage in HUS. To be more specific, the effects of complement-induced damage have been demonstrated in glomerular, primary human umbilical vein, human microvascular and blood outgrowth endothelial cells (21, 26, 28, 30, 31). Although these assays are extremely useful in discerning the various cellular and molecular determinants of CM-HUS pathophysiology, their use as functional assays in the daily routine of a diagnostic laboratory should only be considered in a broader context that also embraces a wide spectrum of genetic analyses and serological or other biochemical assays. Thus, selecting the appropriate functional assays to aid or refine the clinical diagnosis of CM-HUS remains a subject of intense investigation. In this respect, reliable functional assays of APC activation have long been sought after in the field of TMAs. Traditional markers used in clinical complement laboratories, such as hemolytic assays for measuring classical and alternative pathway activity (CH-50 and AP-50, respectively) and Wieslab ELISA for measuring C3 concentration or alternative pathway activity (Wieslab Complement System; Euro Diagnostica, Malmo, Sweden), may yield normal values and thus cannot confirm a diagnosis of CM-HUS (32).

Recently, terminal complement activation products C5a and soluble C5b-9 or membrane attack complex (MAC) were compared in CM-HUS and TTP. In spite of increased plasma C5a and C5b-9 levels in CM-HUS, there was a significant overlap of values between syndromes (33). Other studies have reported urine C5b-9 as a more reliable marker compared to plasma C5b-9 (34, 35). Translational studies have also found increased C5b-9 deposition on human microvascular endothelial cells (HMEC) by confocal microscopy in acute phase and remission of CM-HUS patients compared to controls (36). A most recent study has utilized C5b-9 deposition on HMEC to detect evidence of complement activation in patients with recurrent TMA after transplant (37).

In an effort to develop a rapid and reliable *in vitro* diagnostic assay for CM-HUS, the modified Ham test was introduced based on the principle of the Ham test traditionally used for paroxysmal nocturnal hemoglobinuria (PNH) diagnosis (38). As our understanding of complement-mediated disorders evolves, it seems that cell-based assays may better reflect complement activation *in vitro*. Interestingly, recent studies of CM-HUS associated with mutations in complement-related serum factors reveal that serum complement is not activated *per se*, but activation is caused as a result of defective interaction of complement regulatory proteins, such as factor H, with cell surfaces (39). The modified Ham test utilizes PNH-like cells that are susceptible to complement-mediated cell death to detect complement activated serum, like CM-HUS serum. It has been documented that the modified Ham test distinguishes complement-mediated TMAs from other TMAs (38, 40, 41). Further improvements on the positive control of the modified Ham test and the addition of a confirmatory assay with a strong correlation with the modified Ham test are expected to further improve its accuracy and applicability (42). However, it is a rather cumbersome assay for clinical laboratories and cannot be recommended for clinical routine yet.

Unfortunately, no direct comparison among assays has been performed and therefore, no safe conclusions can be drawn for the usefulness of each assay. Soluble C5b-9 levels, C5b-9 deposits on endothelial cells and the modified Ham test have shown evidence of complement activation in patients with or without mutations or autoantibodies (36, 38, 43). The modified Ham test has also shown a 100% positive predictive value for response to eculizumab (43). However, the modified Ham test and C5b-9 depositions require cell culture techniques, while measurement of soluble C5b-9 has a wider applicability. Standardization is another important issue, especially for cell-based assays. Although the modified Ham test offers a clear cut-off value to differentiate CM-HUS from TTP, soluble C5b-9 levels have a significant overlap between HUS and TTP (33); while no comparison exists for surface C5b-9 deposition (36). In addition, soluble C5b-9 and the modified Ham test have limited applicability on discriminating patients in acute phase vs. remission, although more data on C5b-9 deposition might prove useful in this field. As suggested by initial reports, monitoring and guiding eculizumab treatment may be feasible by measuring C5b-9 deposition on endothelial cells (36) and soluble C5b-9 in the vasculature (35). Lastly, testing the effects of novel complement inhibitors is feasible by employing the modified Ham test (44) and measuring C5b-9 deposition (36).

##### Genetic evidence of complement activation

Genetic testing for CM-HUS is also difficult to utilize in clinical practice. Its major limitations include the high cost and the time consuming process in an urgent life-threatening situation that requires immediate treatment (45). In addition, genetic testing needs to be performed by an expert complement-focused laboratory able to analyze the results of next-generation sequencing (NGS). NGS analysis produces a large number of variants in each patient that needs to be carefully interpreted. Current methodology reports only rare variants, since these are considered to be pathophysiologically linked to the development of rare syndromes such as TMAs. In an effort to better understand the clinical significance of rare variants, helpful databases are created, such as the Database of Complement Gene Variants, that could be an integrated part of bioinformatics analysis in the future (46). However, this approach does not take into account the majority of detected variants, whose functional and clinical significance remains to be studied. Even in this case, genetic testing is useful for about half of the patients with CM-HUS that are expected to harbor a mutation in any of the complement-related genes already implicated in aHUS (27, 47).

##### Pathophysiology of complement activation

CM-HUS is characterized by excessive activation of the alternative pathway of complement (APC) and its pathophysiology is currently described by a two-hit disease model. The first hit results from inherited mutations in APC genes or from acquired alterations in APC activity, such as autoantibodies to APC proteins (anti-CFH antibodies) (5, 48). Commonly associated triggers of the second hit are considered crucial for the manifestation of the disease and include pregnancy, inflammation, surgery or autoimmunity (29, 49, 50).

Mutations cause either loss of function of complement regulatory proteins, including complement factor H (CFH), complement factor I (CFI), thrombomodulin (THBD), or CD46/membrane cofactor protein (MCP), or gain of function of complement activating proteins, including complement factor B (CFB) and C3 (51). Although THBD may also act as a complement regulator (52), further studies are needed to confirm the role of proteins involved in the coagulation pathway (48). A most recent study has also revealed mutations in *VTN*, which encodes the terminal complement inhibitor vitronectin, in CM-HUS patients (17). The only mutations not associated with complement dysregulationre found in diacylglycerol kinase-e (DGKE) (53, 54). Therefore, HUS associated with DGKE mutation is not classified under the terms complement-mediated HUS or HUS with activation of the complement alternative pathway (5, 48).

**Figure 1:**
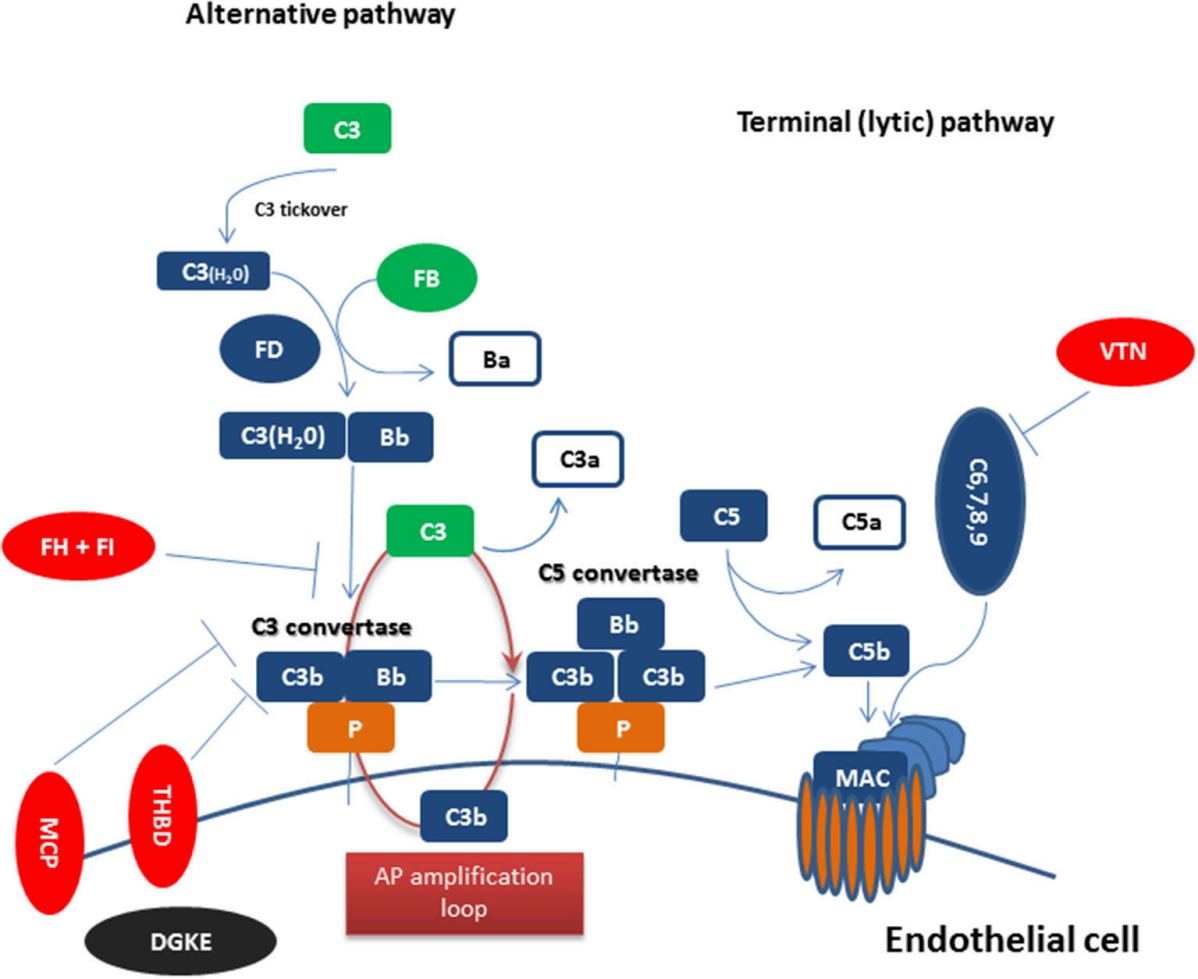
Complement dysregulation in complement-mediated HUS Complement activation initiated by any of the three pathways (classical, alternative, or lectin pathway) leads to C3 activation and C3 convertase formation on C3-opsonized surfaces. C3 activation through the alternative pathway of complement (APC) amplifies this response (APC amplification loop), culminating in pronounced C3 fragment deposition on complement-targeted surfaces (proximal complement). In the presence of increased surface density of deposited C3b, the terminal (lytic) pathway is triggered, leading to membrane attack complex (MAC) formation on the surface of target cells. Dysregulated or excessive complement activation mainly affects renal endothelial cells which show increased susceptibility to complement attack due to a deteriorating glycocalyx in pathologies such as CM-HUS (terminal complement). Complement alternative pathway dysregulation results from loss-of-function mutations in regulatory factors (Factor H, I, THBD/thrombomodulin, and vitronectin/VTN in aHUS) shown in red, gain-of-function mutations (C3 and Factor B) shown in green, and DGKE mutations shown in black, indicating the unknown effect on complement cascade.

Figure 1 summarizes complement dysregulation observed in CM-HUS. Except for rare germline mutations predisposing to CM-HUS, common genetic variants in CFH, CD46, and the CFHRs have been also reported as risk factors for CM-HUS (25). Mutations in CFH related genes occur in up to 5% of the general population (55). Interestingly, genetic mutations are found in 50% of patients diagnosed with CM-HUS, while a number of these mutations has uncertain clinical significance as discussed above.

## Infection-associated Hemolytic Uremic Syndrome (IA-HUS)

### Clinical Features

Infection-associated (IA) or typical or Shiga-toxin-secreting *Escherichia coli* (STEC) HUS represents a TMA of infectious etiology presenting mainly in children infected with Shiga-toxin-secreting *Escherichia coli* 0157:H7. Other subtypes of *E. coli, Salmonella, Shigella*, and *Campylobacter* have been also detected in IA-HUS patients (56). Diagnosis of IA-HUS is confirmed by the presence of an enterohemorrhagic strain of E. coli and/or identification of *Stx1* or *Stx2* genes in the stool sample or rectal swab. Two recent case reports have also identified Bordetella pertussis infection as a trigger of IA-HUS (57, 58).

Clinical manifestations span a wide spectrum from uncomplicated diarrhea to hemorrhagic colitis and post diarrheal HUS. HUS manifestations include MAHA, thrombocytopenia and acute kidney injury, while neurological and cardiac involvement may be also be present in severe forms. Long-term renal involvement has been documented in 30% of surviving patients (59, 60), with mortality rates up to 5% in patients developing HUS (61). Neurologic involvement, anemia, and hyponatremia have been recently described as predictors of mortality in IA-HUS (62).

### Functional and Genetic Evidence of Complement Activation

Evidence from human (63–66) and animal (67–69) studies have suggested that complement activation may play a role in the course of IA-HUS. However, the epidemic nature of the disease hinders functional studies and the role of complement has not been fully characterized. In addition, the largest genetic analysis so far implicates variants mostly in several non–complement-related genes involved in iron transport, cytokine signaling, platelet function, pathogen recognition, and endothelial function and less in complement-related genes (70). However, this analysis focuses on single nucleotide polymorphisms (SNPs) and not on rare variants found in TMAs.

## HELLP Syndrome

### Clinical Features

Preeclampsia is characterized by hypertension and proteinuria, with or without end organ damage occurring in 3–5% of pregnant women (71). HELLP syndrome (hemolysis, elevated liver enzymes, and low platelets) manifests as a severe form of preeclampsia. Since 1982, HELLP syndrome has been reported in up to 0.8% of all pregnancies (72). Diagnostic classifications take into account platelet count, lactate dehydrogenase (LDH) levels, bilirubin and aspartate aminotransferase (AST) with or without alanine aminotransferase (ALT) levels to establish the diagnosis (73, 74). Clinical manifestations resemble CM-HUS, including MAHA, thrombocytopenia, hypertension and renal or neurological dysfunction. Due to these similarities, HELLP syndrome might be often characterized as pregnancy-associated HUS, according to the traditional terminology, and vice versa (75). Of note, HELLP syndrome is a cause of severe morbidity and mortality for both the mother and fetus (76).

### Functional and Genetic Evidence of Complement Activation

The pathophysiology of HELLP syndrome has not been fully elucidated yet, although it has been traditionally considered to be part of the spectrum of preeclampsia (77). Since 1990, Haeger et al. have suggested increased complement activation in HELLP syndrome with increased C5b-9 levels (78). Urine C5b-9 levels have been more recently considered a more robust marker in patients with preeclampsia (34). The modified Ham test has also provided data of increased APC activation in severe preeclampsia and HELLP syndrome. In addition, the modified Ham test was also used as an *in vitro* model suggesting that eculizumab effectively blocks complement-mediated effects in HELLP serum (40). APC-related mutations have been reported in up to 20% of HELLP patients, suggesting that APC dysregulation might be a distinct contributor to HELLP pathology (79). A more recent study has confirmed these findings reporting APC-related rare germline mutations in 46% of patients with HELLP syndrome. The authors were also able to show that combined complement-related phenotypes and genotypes were highly predictive of HELLP syndrome (41).

## Transplant-associated TMA

### Clinical Features

TA-TMA is a potentially life-threatening complication of allogeneic hematopoietic cell transplantation (HCT) observed in 7–39% of HCT recipients (80–85). It manifests with the clinical triad of a TMA including MAHA, thrombocytopenia and often renal or neurologic dysfunction. Its diagnosis is largely hindered by the high incidence of cytopenias and organ dysfunction in HCT recipients. Currently used diagnostic criteria include both the Bone Marrow Transplant Clinical Trials Network (BMT-CTN) and the International Working Group (IWG) criteria (80, 86). Both criteria have been criticized for limitations in their diagnostic sensitivity (87).

### Functional and Genetic Evidence of Complement Activation

Our understanding of TA-TMA pathophysiology is rapidly evolving. Initially, the syndrome was considered and treated as a form of TTP. However, plasma exchange had limited efficacy in these patients (88, 89). In line with these findings, studies have shown that ADAMTS13 is not deficient in TA-TMA and therefore, cannot be used as a disease marker (90, 91). Better understanding of CM-HUS has helped researchers and clinicians understand that TA-TMA resembles more CM-HUS than TTP both in pathophysiological and clinical features (92). This notion is supported by genetic and functional evidence of complement activation in TA-TMA. Jodele et al have first described APC-related mutations in pediatric HCT recipients (93), with additional data of poor prognosis in patients harboring APC-related mutations (94). Data of genetic susceptibility support the idea of the two-hit hypothesis being true also for TA-TMA.

The second hit may result from several clinical factors that have been associated with TA-TMA, including age, donor type, conditioning regimen, calcineurin or mTOR inhibitors, graft-vs. host disease or infections (81, 82, 95–100). Although several cross-sectional associations have been reported, a few reports have investigated underlying mechanisms. Of note, complement dysregulation has been implicated in GVHD regulation of mice (101–103) and humans. Additional links between GVHD and complement activation have been shown in human cutaneous tissues, where C3 inhibition by compstatin reduced CD4^+^ T-cell proliferation and Th1/Th17 polarization (104). A recent *in vitro* study has also implicated the C5a/C5aR IL-17A axis in chronic GVHD (105). Furthermore, C3 levels have been associated with sclerotic cutaneous GVHD patients (106) and patients with sclerotic GVHD have shown abnormalities in complement factor H and APC functional assays (107). Complement activation has been also linked with thrombin generation in patients treated with antithymocyte globulins (ATG) (108).

## Impact of the Products of C3 vs. C5 Convertase Activity in the Context of CM-HUS and Other TMAs

Complement dysregulation at the level of the formation of the C3 and C5 convertases can exacerbate the pathological process by exerting a plethora of detrimental effects that altogether contribute to TMA pathology. For instance, AP amplification leads to pronounced C3 fragment deposition (opsonization) on the endothelial cell wall thus facilitating the recruitment of innate immune cells such as macrophages or monocytes bearing CR3/CR4 phagocytic receptors (109, 110). Additionally, upregulation of P–selectin on endothelial cells in CM-HUS kidneys, may provide a tether for focusing AP convertases and further fueling the AP amplification loop on C3-opsonized endothelial cells (68). Notably C3 can serve as direct binding target for heme released from erythrocytes by mechanical hemolysis within the renal microvascular network (29, 111). Heme has been shown to intercalate into C3 molecules promoting homophilic C3 interactions and subsequent formation of overactive C3/C5 convertases (29). Moreover, the release of C3a in the vicinity of the opsonized endothelium may stimulate the C3aR-dependent activation and recruitment of neutrophils, basophils or mast cells that can cause endothelial damage through degranulation and release of free radicals and proinflammatory mediators (112, 113). Furthermore, C3aR stimulation on the renal endothelium in a murine model of STEC-HUS has been linked to increased thrombogenic responses that can facilitate microthrombi formation and vaso-occlusion (68). Additionally, glomerular endothelial cells appear to be particularly sensitive to complement C3a responses via upregulation of C3aR expression in response to various inflammatory stimuli, thus providing an alternate route for C3aR-dependent endothelial cell activation and damage (16). On the other hand, an overactive C5 convertase may lead to massive release of C5a, a highly proinflammatory mediator that, through neutrophil activation and chemotactic recruitment, can exacerbate endothelial damage (114). Notably, C5a can also promote a thrombogenic response via tissue factor (TF) upregulation and release from either infiltrating monocytes/neutrophils or the renal endothelium itself (26, 115). Furthermore, C5a-mediated stimulation of microvascular endothelial cells may also result in increased cell retraction, promoting paracellular permeability (116). The terminal product of the lytic pathway, C5b-9 (MAC) is known for its capacity to trigger endothelial activation, promoting neutrophil adhesion via P-selectin and ICAM-1 upregulation, and it also enhances vascular permeability, via endothelial cell contraction and gap formation (117, 118). Targeting the complement cascade at the level of the C3 convertase may attenuate endothelial C3b opsonization and thus prevent the adhesion of damaged red blood cells and activated platelets to the endothelium via tethering to neutrophil-expressed CD11b/CD18 (119). Therapeutic C3 inhibition may also reduce the release of C3a, thus blunting its procoagulant activities in the microvascular network of the kidney. Of note, C3 blockade can also prevent the downstream generating of C5-derived inflammatory effectors (C5a, C5b-9), thereby offering a much broader inhibitory strategy for ameliorating pathological changes in the context of CM-HUS and other complement-related TMAs (120).

## Complement Therapeutics

### CM-HUS

#### Current Treatment

CM-HUS is an urgent life-threatening syndrome requiring prompt initiation of therapy. Plasma exchange should be initiated at presentation, often before the results of differential diagnosis are available due to the aggressiveness of the syndrome. Although plasma exchange is effective in some patients underlying complement-mediated damage to kidneys and central nervous system often persists (121). Within 1 year from diagnosis, more than 50% of patients treated with plasma exchange or plasma infusion develop permanent renal damage, progress to end-stage renal disease or die (122). Affected patients may suffer from lifelong systemic complications causing multiple organ damage of (renal, gastrointestinal, central nervous system, cardiac) and death.

In recent years, clinical complement intervention has revolutionized the field, with a diverse array of complement-targeted drug candidates currently being evaluated in clinical trials as treatment options for several complement-mediated indications (123). The first-in-class complement inhibitor, eculizumab, a complement C5-targeting monoclonal antibody that blocks generation of C5a and prevents the assembly of the pore-forming MAC, marked a milestone in therapeutic complement inhibition, showing efficacy and safety in two prospective clinical trials of primary adult CM-HUS patients (15, 124). These studies led to the FDA approval of eculizumab for the treatment of atypical HUS in 2011. Eculizumab has shown high efficacy with sustained benefits in 2-year follow-up data and good safety profile in both children and adults (124–126). More recent open label studies have also shown high efficacy rates of approximately 70% and no eculizumab related death (127–129). Although experience with eculizumab raised confidence in the approach, its clinical use in aHUS has revealed limitations that warrant further investigation.

Given the lack of a confirmatory diagnostic assay and the high cost of the drug, therapy is often delayed or not administered. Commonly used criteria of plasma exchange failure include: (1) failure to achieve hematologic response (improvement in platelet count and decrease in the LDH) over the first 4–5 days, (2) progressive end organ injury (renal and/or neurologic) over the first 4–5 days of PEX therapy, and (3) ADAMTS13 activity higher than 10% (130, 131). However, clinical application of these criteria is not always straightforward. Even in CM-HUS patients, mild hematologic response is often observed after 4–5 plasma exchange sessions, leading to clinical dilemmas for treating physicians. The fact that response to eculizumab is often used to confirm CM-HUS diagnosis is indicative of the diagnostic difficulties in the disease (132). Another important issue for clinicians is the risk of Neisserial infections due to terminal complement blockade. Thus, all patients treated with eculizumab should receive anti- meningococcal vaccination at least 2 weeks before initiation of treatment. In severe life-threatening syndromes, such as TMAs, experience from PNH patients has shown that vaccination and eculizumab can be administered the same day along with 2 weeks of prophylactic treatment with ciprofloxacin (133). In immunocompromised patients, such as those with transplant-associated TMA, administration of eculizumab has been safe and effective without anti-meningococcal vaccination (134).

Last, accumulating evidence suggests that cessation of treatment is feasible in the majority of patients. Studies of eculizumab cessation have enrolled 91 patients. Among them, 27 (approximately 30%) relapsed (135–139). The majority of patients had a known mutation. Recurrences have been also reported in individual patients: 1 with complement factor H mutation (140), 1 with C3 mutation (141), and 1 after kidney transplantation (142). In all cases, close patient monitoring led to prompt re-initiation of treatment and complete recoveries. An alternative strategy toward restrictive use of eculizumab includes prolongation of time intervals between dosages (143). However, this requires either therapeutic drug monitoring or measuring of the pharmacodynamics effect that are not available in all centers. Further studies are needed to determine the high-risk patient population prone to relapse or a widely applicable assay for monitoring patients. Until then, close surveillance for signs and symptoms of recurrent TMA is recommended if physicians and patients decide to discontinue eculizumab.

#### Next-Generation Complement Therapeutics

A plethora of novel complement inhibitors is on the horizon, with some being in late clinical development (123, 144, 145). Table 1 summarizes novel complement inhibitors currently being evaluated in pre-clinical and clinical phases. The choice of the appropriate target and drug has to be carefully considered taking into account the limitations of eculizumab treatment. To accomplish that, an important question needs to be answered. Is there a need for novel complement inhibitors in CM-HUS?

**Table 1:** Complement-targeted therapeutics in various stages of clinical development for complement-mediated indications.

**Complement therapeutic**	**Entity**	**Target**	**Mechanism of action**	**Mode of administration**	**Pharmaceutical sponsor**	**Stage of development**
Eculizumab^*^	mAb	C5	Inhibition of C5 activation	IV infusions	Alexion Pharmaceuticals	In the clinic
ALXN1210/ ravulizumab^*^	mAb	C5	C5 inhibition/same epitope as ecu	Bimonthly IV infusions	Alexion Pharmaceuticals	Phase II/III
ABP959	mAb	C5	Inhibition of C5 activation/biosimilar of ecu	IV infusions	Amgen	Phase III
SKY59/RO7112689	mAb	C5	Inhibition of C5 activation/different epitope from ecu	IV and SC injections	Hoffmann-La Roche	Phase I/II
LFG-316/ tesidolumab	mAb	C5	Inhibition of C5 activation/different epitope from ecu	IV infusions	Novartis	Phase II
REGN3918	mAb	C5	n.a.	IV and SC	Regeneron	Phase I
Mubodina	Minibody (Fab- based)	C5	C5 inhibition/ different epitope from ecu	n.a.	Adienne	Preclinical stage
Coversin (OmCI)	Recomb. protein	C5	Inhibition of C5 activation	SC injections	Akari Therapeutics	Phase II
RA101495	Peptide	C5	Allosteric inhibition of C5 activation	Daily SC injections	Ra Pharmaceuticals	Phase II
Cemdisiran (ALN-CC5)^*^	siRNA	C5	Silencing of hepatic C5 production	SC injections	Alnylam	Phase II
AMY-101	Peptide	C3	C3 inhibition/ blockage of C3 convertase activity	Daily SC injections	Amyndas Pharmaceuticals	Phase I completed/Phase II announced
APL-2	PEGylated peptide	C3	C3 inhibition/ blockage of C3 convertase activity	Daily SC injections	Apellis Pharmaceuticals	Phase II, (Phase III announced)
Mini-FH/AMY-201	Recomb protein	AP C3 convertase	Surface directed inhibition of AP	n.a.	Amyndas Pharmaceuticals	Preclinical stage
LNP023	Small Molecule	Factor B	Inhibition of AP C3 convertase formation	Orally	Novartis	Phase II
IONIS-FB-LRx	Antisense oligonucleotide	Factor B	Inhibition of AP C3 convertase formation	SC injections	Ionis Pharmaceuticals/Roche	Phase II (announced)
ACH-4471/ACH-0144471	Small molecule	Factor D	Inhibition of AP C3 convertase formation	Orally	Achillion Pharmaceuticals	Phase II
Lampalizumab	mAb (Fab)	Factor D	Inhibition of AP C3 convertase	Intravitreal inj.	Genentech/Roche	Phase III (discontinued)
CLG561 (Novartis)	mAb	Properdin	Inhibition of Alternative pathway	Intravitreal injections	Novartis	Phase II
TNT009/BIVV009/ Sutimlimab	mAb	C1s	CP inhibition/inhibition of C1s protease activity	IV infusions	True North Therapeutics/Bioverativ/Sanofi	Phase II/III (CAD patients)
OMS721	mAb	MASP-2	Inhibition of Lectin P activation	IV	Omeros corporation	Phase III
Mirococept (APT070)	Recomb. protein	C3/C5 convertases	Inhibition of both CP and AP convertases	IV	MRC (UK)	Phase III
Avacopan (CCX168)^*^	Small molecule	C5aR1	Inhibition of C5aR1 signaling	Orally	Chemocentryx	Phase III
IFX-1	mAb	C5a	Blocks biological activity of C5a	IV	(InflaRx)	Phase II

IV, intravenous; SC, subcutaneous; CP, classical pathway; AP, alternative pathway; siRNA, small interfering RNA; n.a., not available;

* *Components in clinical development for complement-mediated TMAs*.

Unlike PNH, the medical community has failed to recognize a specific sub-group of CM-HUS patients that are likely to benefit from novel complement inhibitors. Eculizumab's limitations include the requirement of life-long intravenous infusions every 2 weeks and the substantial economic burden associated with chronic anti-C5 treatment. To overcome these limitations, a number of novel C5 inhibitors have been introduced that are either long-lasting or administered in a subcutaneous or oral form. Such inhibitors are currently investigated in CM-HUS patients: Cemdisiran (an investigational RNAi therapeutic targeting complement component C5 administered subcutaneously every 4 weeks), ALXN1210 (a longer-lasting intravenously administered C5 inhibitor) and Avacopan (an orally-administered small molecule inhibitor of C5a receptor 1), as highlighted in Table 1.

From the pathophysiological point of view however, C5 inhibition with alternative agents is not expected to lead to significant advantages in efficacy and safety compared to eculizumab. Therefore, alternative targets in proximal complement pathways that efficiently inhibit the alternative complement pathway involved in HUS may provide advantages in HUS patients not only in terms of higher efficacy but also in terms of potentially less infectious complications. Although the effects on infectious complications need to be further studied, early data suggest that inhibitors of the alternative pathway leave the classical and lectin pathways active against invasive pathogens (146). Furthermore, it should be noted that the opsonophagocytic killing of meningococci in whole blood from vaccinated volunteers was recently shown to be increased in the presence of an alternative pathway-directed inhibitor (anti-FD agent, ACH-4471) as compared to anti-C5 treatment, thereby suggesting that vaccination may provide better protection against meningococcal disease in patients treated with an AP-specific inhibitor (147). Targets of the alternative pathway may include factors B, D, and the alternative pathway C3 convertase. Inhibitors of these agents under development for complement-mediated indications are summarized in Table 1. Both factor B inhibitor LPN023 and factor D inhibitor ACH-4471 have the additional benefit of oral administration which is particularly useful for lifelong administration. Both inhibitors are currently under phase II clinical trials: factor B inhibitor in IgA nephropathy and PNH, factor D inhibitor in C3G nephropathy and PNH.

Recently, the clinical advancement of C3-targeted inhibitors of the compstatin family has opened up new avenues for exploring viable anti-complement therapies in various clinical indications, including complement-mediated TMAs (148). Of note, the C3-targeted peptide therapeutics APL-2 and AMY-101, both developed on the same compstatin scaffold with various modifications aimed at increasing plasma residence and target affinity, are being evaluated in Phase Ib-III trials in clinical indications ranging from PNH and autoimmune hemolytic anemias to geographic atrophy and C3 glomerulopathies (123, 145). Thus, far, C3-targeted therapeutic agents have displayed safety, tolerability, preliminary biological efficacy and a capacity to saturate plasma C3 levels during prolonged dosing in humans, thus supporting the investigational use of C3 inhibitors in human clinical trials. By affording broader and comprehensive inhibition of the complement cascade, regardless of initiating trigger or pathway, C3 inhibitors can simultaneously intercept multiple pathogenic drivers in complement-mediated TMAs. Of note, the contextual nature of C3's involvement in pathogen immunosurveillance and bacterial outgrowth (149), the ability to swiftly recover C3 activity in plasma after interrupting treatment with small-sized C3 inhibitors (as compared to the slower plasma clearance of larger biologics) and the likely auxiliary role of C3 in immunosurveillance during adulthood, as supported by observations in younger patients with primary complement deficiencies (150), all argue against the long-held assertion that prolonged pharmacological C3 intervention might increase the risk of infections in treated patients. Furthermore, the stable immune and blood biochemical profile of non-human primates (NHP) subjected to prolonged, systemic C3 inhibition, together with the faster skin wound healing and absence of skin infections in NHPs treated with the C3 inhibitor Cp40, further attest to the safety of this targeting approach (151).

In conclusion, whether inhibitors of the alternative pathway of complement can offer an effective and safe treatment in complement-mediated TMAs will be ultimately determined in future clinical trials. To prove the efficacy of novel complement inhibitors, experimental models of complement activation might be a useful step before clinical application (44).

### IA-HUS

The care of children with IA-HUS remains supportive, with no established targeted therapies. Debated approaches include plasma exchange, plasma infusions, immunoadsorption and antibiotics. Terminal complement inhibition by eculizumab has also been used in IA-HUS with controversial results and reports suggesting some benefit (152, 153), while others stating no benefit (56, 154). A large multicenter retrospective study has recently tried to shed light on the role of terminal complement inhibition (155). Despite limitations linked to the nature of the syndrome and methodology, this study suggested that eculizumab is effective in patients with neurological dysfunction and patients with sustained complement inhibition. In addition, experimental evidence of the driving role of C3-mediated thrombosis in IA-HUS suggest that proximal complement inhibition at the level of C3 might provide additional advantages in these patients (68). Indeed, the beneficial effect of C3aR antagonism on attenuating thrombogenic responses in a rodent model of STEC-HUS indicates that upstream complement intervention at the level of C3 might offer broader therapeutic coverage in IA-HUS. C3 inhibitors could likely interfere with multiple pathogenic drivers in IA-HUS by simultaneously blocking the generation of C3-derived proinflammatory effectors (i.e., C3a) and also by attenuating the generation of terminal (lytic) pathway effectors that contribute to microvascular endothelial injury and inflammation (i.e., C5a, C5b-9) (68). It is noteworthy that a broader role for C3a-C3aR signaling in modulating thrombogenic responses (i.e., NETosis-driven hypercoagulation) has been documented in diverse pathologies including intestinal cancer (156). Taking into account these considerations, prospective controlled studies are expected to provide more insight into the role of complement inhibition in IA-HUS (Table 1).

### HELLP

Current treatment strategy remains supportive in HELLP syndrome, consisting of steroid and magnesium administration and proper hypertension management (71, 157, 158). The treatment of choice is delivery, taking into account that neonatal morbidity and death are mainly associated with gestational age (158). The first successful use of targeted anti-complement treatment (i.e., eculizumab) was reported in 2013, suggesting that eculizumab permits safe prolongation of pregnancy and successful outcomes for both the mother and the fetus (159). Indeed, eculizumab's use in PNH patients has proven safety and efficacy during pregnancy (160). Nevertheless, further studies are needed to explore complement inhibition in HELLP syndrome. Of note, the frequent presence of germline APC gene mutations in HELLP patients, along with a pronounced activation of the APC in patient sera (41), both suggest that therapeutic modulation of the AP, or targeted inhibition of its central protein C3, might be a promising new avenue to alleviate pathology in these patients.

### TA-TMA

The majority of TA-TMA patients are refractory to conventional treatment, leading to high mortality rates (up to 100%, median 75%) (161). Depending on each clinical center's policy conventional treatment includes withdrawal of calcineurin inhibitors, corticosteroids, plasma exchange or rituximab. Novel approaches have investigated eculizumab treatment in both adult and pediatric patients with TA-TMA (134, 162–164). Although results are encouraging compared to mortality rates in the pre-eculizumab era, timing of initiation, proper patient selection, dosing and duration of therapy remain to be further investigated in this complex field of transplanted patients. In light of the emerging correlation of TA-TMA pathology with distinct genetic aberrations in the APC, and the central role of C3 in amplifying APC activation, it is intriguing to speculate that therapeutic strategies targeting upstream complement components, or centrally the C3 protein, may elicit more beneficial therapeutic outcomes in TA-TA patients. Therefore, the clinical evaluation of novel complement-based drug candidates that are registered in the pipelines of several biopharmaceutical companies (see Table 1), is highly anticipated in patients with TA-TMA. Despite lack of pathophysiological evidence suggesting activation of the lectin pathway, an inhibitor of the lectin pathway, the MASP-2 inhibitor OMS721, has shown encouraging results in a preliminary analysis of a phase II study in TA-TMA patients (Table 1). However, these results were compared to dramatic outcomes of a historical control group treated only by conventional treatment.

## Conclusion

Complement-mediated TMAs represent a rapidly evolving field aiming to provide better outcomes for patients with benign, yet life-threatening syndromes. Unraveling Ariadne's thread into the labyrinth of complement therapeutics is challenging, especially when novel agents are continuously studied and novel patient groups are identified. Beyond TMAs analyzed in this review, preliminary data suggest complement dysregulation in more entities, such as TMAs associated with lupus nephritis (165). Better understanding of the role of complement dysregulation in these entities will facilitate diagnosis, promote patient stratification into cohorts that may optimally respond to therapeutic modulation of the complement system and provide effective therapeutic options for treating physicians.

## Author Contributions

EG and DM conceived the review topic, wrote the outline, and the manuscript. AA edited and approved the manuscript.

### Conflict of Interest Statement

The authors declare that the research was conducted in the absence of any commercial or financial relationships that could be construed as a potential conflict of interest.
